# A Rare Case of Acute Aortic Occlusion Mimicking Cauda Equina Syndrome in a Patient With Inflammatory Bowel Disease

**DOI:** 10.7759/cureus.73938

**Published:** 2024-11-18

**Authors:** Ana Raquel Soares, José Ganicho, Sofia Eusébio, Paula Nascimento, Tiago F Ribeiro

**Affiliations:** 1 Internal Medicine, Unidade Local de Saúde São José, Lisbon, PRT; 2 Infectious Diseases, Unidade Local de Saúde São José, Lisbon, PRT; 3 Vascular Surgery, Unidade Local de Saúde São José, Lisbon, PRT

**Keywords:** acute lower limb ischemia, embolus, inflammatory bowel disease, neurogenic bladder dysfunction, neurogenic bowel dysfunction, saddle aortic embolism, spinal cord ischemia

## Abstract

Acute aortic occlusion (AAO) is a rare and life-threatening condition, mostly secondary to acute thrombosis or embolism. It usually presents as bilateral lower limb ischemia; however, in rare cases, spinal cord infarction might coexist, mimicking cauda equina syndrome. We present a rare case of AAO by saddle embolism of a thoracic aortic mural thrombus.

A 48-year-old male patient with a past medical history of ulcerative colitis and smoking was admitted with urinary and fecal incontinence, flaccid paraplegia, bilateral lower limb anesthesia, peripheral cyanosis, and pulseless lower extremities. One month earlier, he had been hospitalized due to an acute inflammatory bowel disease flare. A computed tomography (CT) angiography performed during this hospitalization revealed a pedunculated thrombus in the descending thoracic aorta, which was not present in previous imaging scans. Considering these findings, the hypothesis of acute infrarenal aortic saddle embolism of the previously identified thoracic aortic thrombus was raised and confirmed by CT angiography. Anticoagulation was immediately initiated, and the patient was submitted to emergent surgical revascularization by surgical thrombo-embolectomy with Fogarty catheters. After surgery, limb perfusion improved and the patient’s neurologic deficits progressively resolved. The hypercoagulable state conferred by inflammatory bowel disease (IBD) was considered the underlying mechanism for arterial thromboembolism since other etiological investigations were negative.

This case highlights the importance of recognizing atypical manifestations of AAO and reinforces the prognostic impact of early diagnosis and surgical referral since later intervention presents elevated mortality and morbidity risk. It also demonstrates the probable relationship between IBD and arterial thromboembolism risk, constituting a rare case of AAO associated with IBD.

## Introduction

Acute aortic occlusion (AAO) is a rare, life-threatening condition, which is estimated to occur in 2.7-5.0 cases per one million person-years [1]. It primarily affects older adults with cardiovascular comorbidities [2]. AAO refers to blood flow obstruction through the aorta due to acute thrombosis or embolism, leading to ischemia of the downstream tissues [2,3]. Contemporary data estimate that approximately 20% of these cases consist of saddle aortic embolism [1], which presents a higher mortality [3]. AAO is associated with high morbidity and mortality rates [2]. A recent 16-year retrospective cohort study estimates an overall 30-day mortality rate of 23% [4].

This condition is usually characterized by bilateral acute lower limb ischemia with rhabdomyolysis and ischemic peripheral nerve damage, leading to limb anesthesia and paralysis. In rare cases, spinal cord infarction might coexist, mimicking cauda equina syndrome, in the absence of medullary compression [5,6].

Risk factors for AAO include coagulopathy, smoking, cardiac or pulmonary disease, hypertension, diabetes mellitus, cancer history, prior vascular surgery, and chronic kidney disease [2]. AAO caused by arterial embolism typically occurs in the setting of atrial fibrillation, ventricular thrombus, heart failure, valvular disease, and, rarely, tumor embolism [2].

We present a rare case of AAO by saddle embolism secondary to the hypercoagulable state conferred by inflammatory bowel disease (IBD).

## Case presentation

A 48-year-old male patient presented to the emergency department with a three-hour history of lower limb pain and reduced strength rendering him unable to stand, followed by fecal and urinary incontinence. His medical history was notable for active smoking and a 25-year history of ulcerative colitis. He did not adhere to regular surveillance and remained medicated with prednisone and azathioprine for several years.

**Figure 1:**
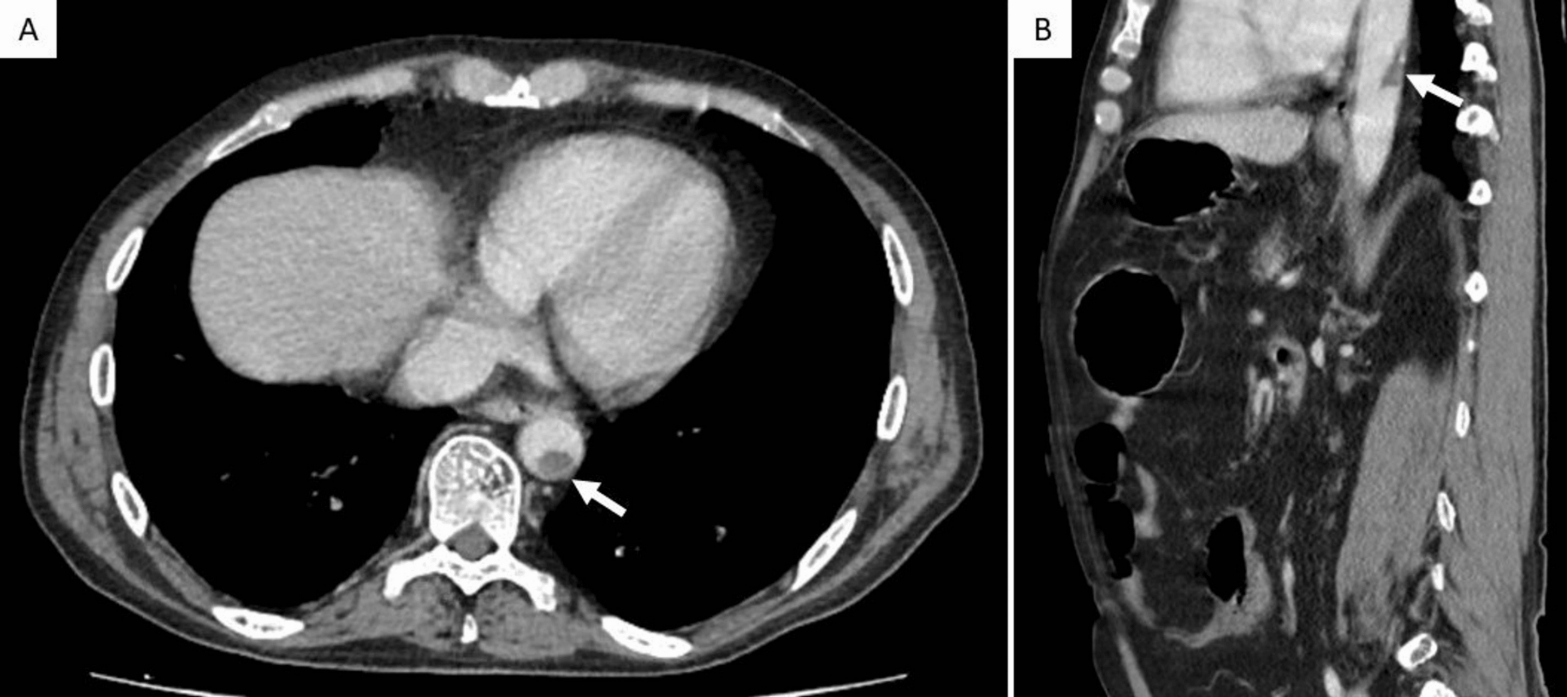
Thoracic aortic pedunculated thrombus in a computed tomography angiography performed one month prior (white arrows) Axial view (A) and sagittal view (B)

One month prior, he had been hospitalized due to an acute IBD flare with suspected associated infective colitis for which he was treated with antibiotic and corticosteroid therapy. At that time, computed tomography (CT) angiography revealed a 16-mm pedunculated thrombus in the descending thoracic aorta (Figure 1), without compromising blood flow.

**Figure 2:**
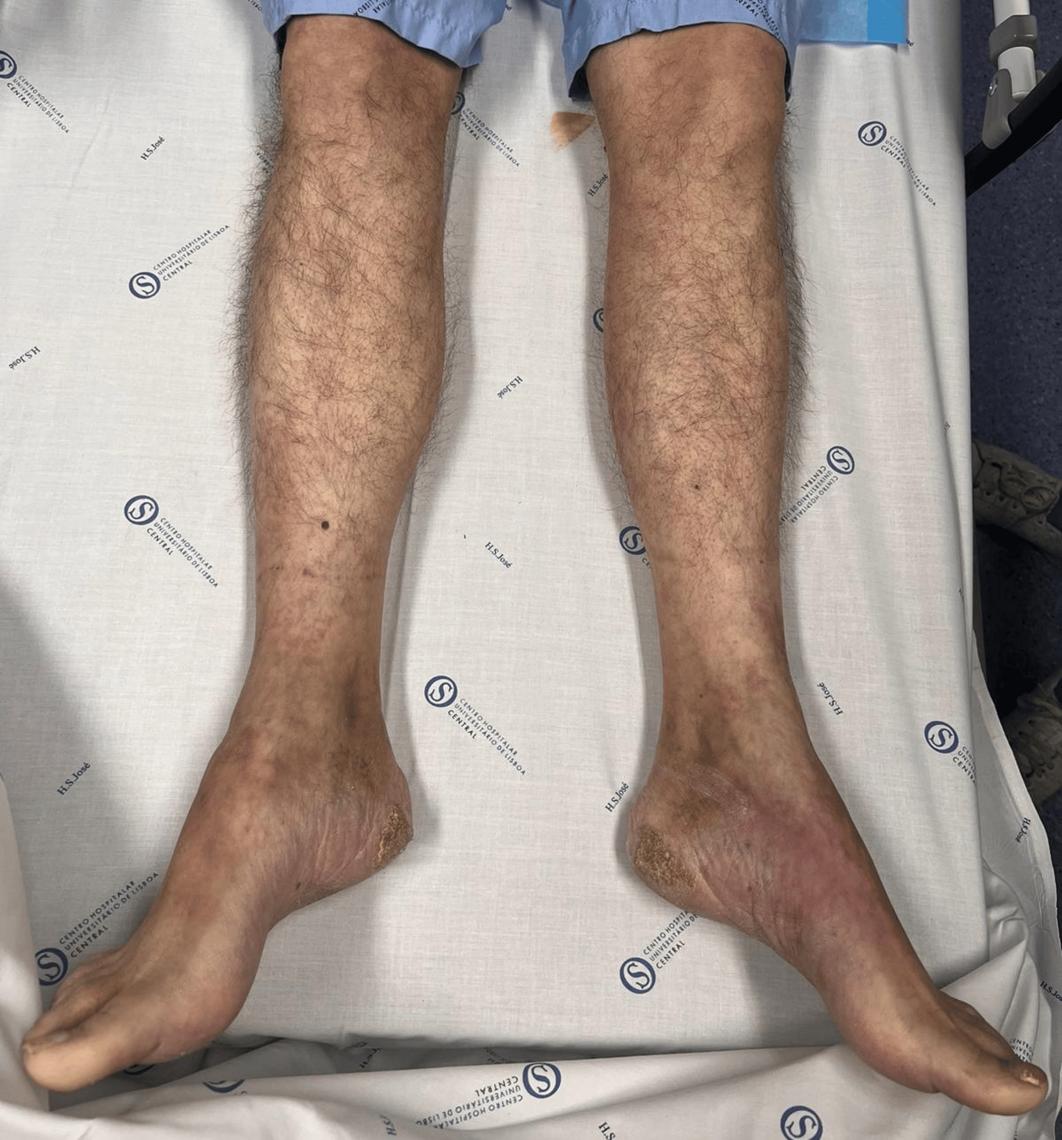
Lower limbs presentation with paraplegia, anesthesia, and peripheral cyanosis

In his current presentation, he exhibited urinary and fecal incontinence, flaccid paraplegia, bilateral lower limb anesthesia (loss of proprioception, tactile sensation, and temperature perception), peripheral cyanosis, and pulseless lower extremities (Figure 2). Physical examination was otherwise unremarkable. Laboratory tests revealed elevated D-dimer levels (2089 μg/L), C-reactive protein (69 mg/L), fibrinogen (6.6 g/L), and creatine kinase (698 U/L) while all other parameters were within the normal range.

**Figure 3:**
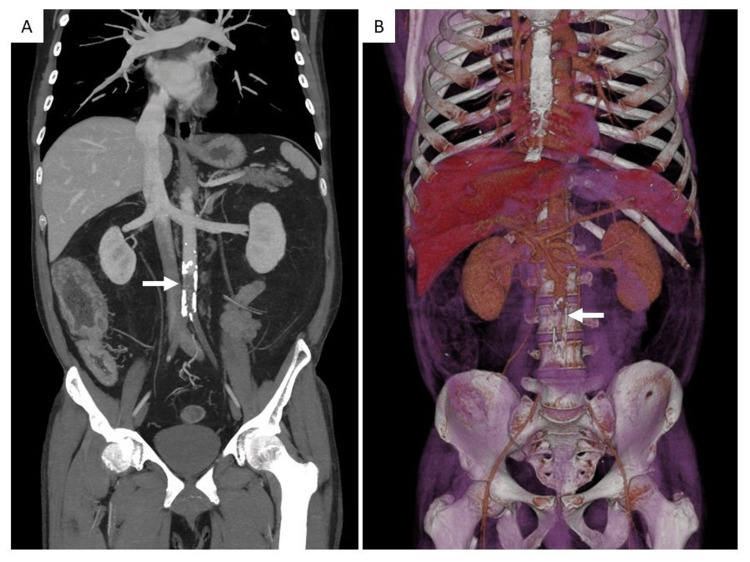
Acute abdominal aortic embolus below the inferior mesenteric artery (white arrows) Coronal view (A) and three-dimensional volume-rendering reconstruction (B)

Considering all these findings, the hypothesis of acute infrarenal aortic saddle embolism of the previously identified thoracic aortic thrombus, resulting in bilateral acute lower limb and spinal cord ischemia, was raised and confirmed by CT angiography (Figure 3). Other causes associated with similar clinical presentation were excluded by imaging, namely, compressive or infiltrative spinal conditions that can cause cauda equina syndrome. Anticoagulation with unfractionated heparin was immediately initiated, and the patient was then submitted to emergent surgical revascularization, by surgical thrombo-embolectomy with Fogarty catheters, without peri-operative complications.

Following surgery, limb perfusion improved and the patient’s neurologic deficits progressively resolved, regaining sphincter control and the ability to walk autonomously, persisting a decrease in distal sensitivity of the lower limbs.

Histological examination of the aortic embolus revealed a typical clot, with no evidence of neoplasm or microorganisms. No genetically determined nor acquired hypercoagulable condition was found. During hospitalization, upon detailed physical examination, a suspicious lesion on the scalp was identified. After evaluation by dermatology and a biopsy of the lesion, basal cell carcinoma was confirmed. Metastatic disease was excluded, and the patient was successfully submitted to surgical excision with complete tumor removal.

As no other conditions associated with arterial thromboembolism (ATE) were found and basal cell carcinoma was localized, this event was considered secondary to the hypercoagulable state conferred by IBD.

As recommended for similar cases, the patient remained anticoagulated after surgery and was discharged on apixaban. Regarding IBD, after evaluation by gastroenterology with a case review and complementary study, considering he presented moderate to severe disease, therapy with ustekinumab was initiated to induce remission, successfully.

## Discussion

This scenario highlights that in all cases of lower limb paresthesia and paralysis with or without pain, a neurovascular examination, including assessment of pulses and skin changes to avoid misdiagnosis, is essential. Presentation with urinary and fecal incontinence can be easily mistaken for a neurologic etiology.

The first-line imaging modality in patients with suspected AAO is thoracic, abdominal, and pelvic CT angiography [2]. Once diagnosed, the management cornerstone for AAO patients is the initiation of anticoagulation, which reduces the risk of ongoing embolism or thrombus progression (secondary thrombus) [2], as performed in this case. Other core components of AAO management include maintenance of normal oxygen saturation, intravenous fluid resuscitation targeting euvolemia, and pain control [2].

A relative decline in open aortic surgery for AAO and the increased use of hybrid and purely endovascular surgical techniques has been noted and might be associated with a 30-day mortality decline in recent years [4]. Regarding the surgical treatment modality, direct revascularization should be recommended [4], as was performed in this case, with a favorable outcome.

After investigation for thromboembolism etiology, no causes other than the hypercoagulable state conferred by IBD were found, considering that localized basal cell carcinoma has not been referred to as a risk factor for arterial thromboembolism. The risk of developing thromboembolism in patients with IBD increases two to three-fold [7]. Potential mechanisms involve chronic inflammation, oxidative stress, endothelial dysfunction, hypercoagulability, abnormal platelet function, gut dysbiosis, and drug-related side effects [8]. ATE is less common than venous events in these patients and incidence is more common after interventional procedures, but spontaneous ATE cases have been reported in patients with IBD [7,9]. Several factors associated with IBD increase the risk of thromboembolic events such as the severity of the disease, hospitalization, surgery, and corticosteroids [7]. Considering that this patient was on systemic glucocorticoid therapy for almost eight years, this could have contributed to the thromboembolic risk. Prophylaxis for thromboembolic events is recommended for patients hospitalized with IBD flare-ups but there is no evidence regarding its continuation after discharge [7,10]. Future studies regarding prophylaxis for thromboembolic events as well as the type and duration of therapeutic anticoagulation in patients with IBD are needed.

## Conclusions

This case highlights the importance of prompt diagnosis and early surgical referral in AAO. Delayed intervention presents higher mortality and the risk of permanent sequelae, namely, amputation and neurologic deficits. It also demonstrates the importance of recognizing atypical manifestations of this unusual condition related to spinal cord ischemia. 

Also remarkable is the probable relation between inflammatory bowel disease and arterial thromboembolism. Cases of AAO associated with IBD were rarely reported, reinforcing the importance of sharing these situations.
